# Infected aortic aneurysm caused by *Helicobacter cinaedi*: case series and systematic review of the literature

**DOI:** 10.1186/s12879-020-05582-7

**Published:** 2020-11-17

**Authors:** Takahiro Matsuo, Nobuyoshi Mori, Atsushi Mizuno, Aki Sakurai, Fujimi Kawai, Jay Starkey, Daisuke Ohkushi, Kohei Abe, Manabu Yamasaki, Joji Ito, Kunihiko Yoshino, Yumiko Mikami, Yuki Uehara, Keiichi Furukawa

**Affiliations:** 1 Department of Infectious Diseases, St. Luke’s International Hospital, 9-1, Akashi-cho, Chuo-ku, Tokyo, Japan; 2 Department of Cardiology, St. Luke’s International Hospital, Tokyo, Japan; 3 Penn Medicine Nudge Unit, University of Pennsylvania, Philadelphia, USA; 4 Leonard Davis Institute for Health Economics, University of Pennsylvania, Philadelphia, USA; 5 Department of Infectious Diseases, Fujita Health University, Aichi, Japan; 6 St. Luke’s International University Library, Tokyo, Japan; 7 Department of Diagnostic Radiology, Division of Neuroradiology, Oregon Health & Science University, Portland, OR USA; 8 Department of Infectious Diseases, Cancer Institute Hospital, Japanese Foundation for Cancer Research, Tokyo, Japan; 9 Department of Cardiovascular Surgery, St. Luke’s International Hospital, Tokyo, Japan; 10 Department of Cardiovascular Surgery, Tokyo Bay Urayasu Ichikawa Medical Center, Chiba, Japan; 11 Department of Clinical Laboratory, St. Luke’s International Hospital, Tokyo, Japan; 12 Department of Infectious Diseases, Asahi General Hospital, Chiba, Japan

**Keywords:** *Helicobacter cinaedi*, Infected aneurysm, Japan, Case report

## Abstract

**Background:**

*Helicobacter cinaedi* is rarely identified as a cause of infected aneurysms; however, the number of reported cases has been increasing over several decades, especially in Japan. We report three cases of aortic aneurysm infected by *H. cinaedi* that were successfully treated using meropenem plus surgical stent graft replacement or intravascular stenting. Furthermore, we performed a systematic review of the literature regarding aortic aneurysm infected by *H. cinaedi*.

**Case presentation:**

We present three rare cases of infected aneurysm caused by *H. cinaedi* in adults. Blood and tissue cultures and 16S rRNA gene sequencing were used for diagnosis. Two patients underwent urgent surgical stent graft replacement, and the other patient underwent intravascular stenting. All three cases were treated successfully with intravenous meropenem for 4 to 6 weeks.

**Conclusions:**

These cases suggest that although aneurysms infected by *H. cinaedi* are rare, clinicians should be aware of *H. cinaedi* as a potential causative pathogen, even in immunocompetent patients. Prolonged incubation periods for blood cultures are necessary for the accurate detection of *H. cinaedi*.

## Background

*Helicobacter cinaedi* is a gram-negative spiral rod that was first discovered in the rectal culture from a man who had had sex with a man with proctitis [1]. *H. cinaedi* was thought to cause infection only in immunocompromised individuals; however, it has also been observed as a causative pathogen in immunocompetent patients [2–4]. *H. cinaedi* can cause bacteremia, skin and soft tissue infection, and arterial infection [5, 6].

Although infected (mycotic) aortic aneurysms are not common, they are difficult to treat and are associated with high morbidity and mortality. Mortality has been reported to be greater than 20%, usually attributable to delays in diagnosis and subsequent complications, such as rupture and sepsis [7–9]. Common pathogens include *Staphylococcus aureus, Streptococcus pneumoniae*, and non-typhoidal *Salmonella* followed by other gram-negative organisms such as *Escherichia coli, Klebsiella,* and *Pseudomonas* spp. [10, 11]. *Mycobacterium* spp., *Treponema palladium*, and *Chlamydophila* spp. have also been reported as causative pathogens, although rarely [12, 13].

Herein, we report three cases of aortic aneurysm infected by *H. cinaedi* that were successfully treated using meropenem plus surgical stent graft replacement or intravascular stenting. Furthermore, we performed a systematic review of the literature regarding aortic aneurysm infected by *H. cinaedi*.

### Case series

#### Case 1

**Fig. 1:**
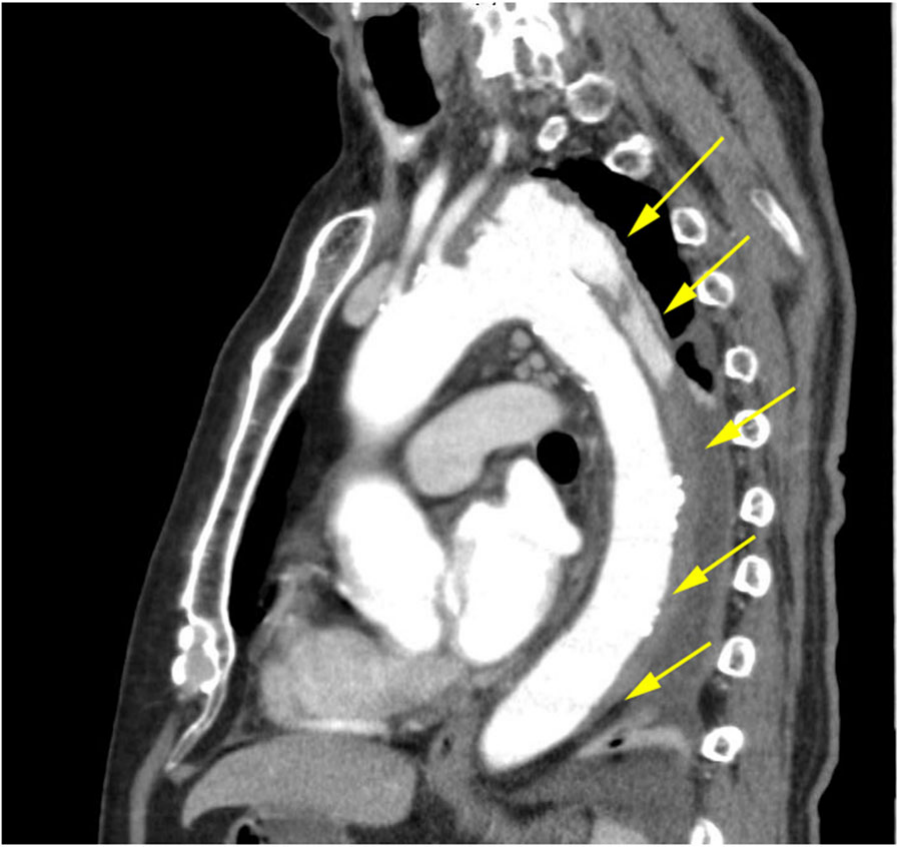
Sagittal contrast-enhanced CT chest-abdomen-pelvis image demonstrates aorta wall thickening with pseudoaneurysm of the distal aortic arch and suggestion of Stanford type B dissection involving the descending aorta, with abnormal fluid extending to the level of the diaphragm

A 77-year-old immunocompetent man with a past medical history of hypertension and dyslipidemia presented to our department with a fever up to 38 °C and progressive left pleuritic chest pain for 2 weeks. On admission, the patient was not in acute distress and had a temperature of 37.6 °C, blood pressure of 143/76 mmHg, heart rate of 67/min, respiratory rate of 16/min, and oxygen saturation of 95% on room air. The patient was noted to have coarse crackles over the left lower lobe of the lung and tenderness of the lower abdomen without any rebound or guarding. Laboratory data showed a mildly elevated white blood cell (WBC) count of 8100/μL and C-reactive protein (CRP) of 15.0 mg/dL. Contrast-enhanced computed tomography (CT) chest-abdomen-pelvis examination demonstrated an aneurysm (30 × 42 mm) of the aortic arch with suggestion of a Stanford B dissection involving the descending aorta on a background of abdominal vessel wall thickening (Fig. 1), increased thickness of bilateral common iliac arteries (20 × 25 mm), and a 1 cm diameter low-density area in the spleen, compatible with infected vasculitis and splenic abscess.

We initiated ceftriaxone 2 g intravenously (IV) every 24 h, vancomycin IV 1 g every 12 h, and minocycline IV 100 mg every 12 h. Due to the active infection, we opted for conservative management without immediate surgery. Despite empirical antimicrobial therapy, the patient had progressive lower abdominal pain on hospital day 4, and a follow-up CT scan demonstrated enlargement of the aortic arch aneurysm (33 × 49 mm) and worsening aortic dissection. Therefore, total arch replacement was urgently performed on the same day. After surgery, the patient gradually improved. On hospital day 7, gram-negative spiral rods were cultured from blood samples obtained on admission (aerobic bottle, BacT/ALERT [bioMérieux, Inc., Durham, NC]). The empirical therapy was changed to meropenem 1 g IV every 6 h. Final blood culture results revealed *H. cinaedi*. The minimum inhibitory concentrations (MICs) measured by Etest (bioMérieux) for this strain were as follows: meropenem 0.008 μg/mL, penicillin G > 32 μg/mL, piperacillin/tazobactam 16 μg/mL, cefotaxime > 32 μg/mL, and levofloxacin > 32 μg/mL. Tissue culture of the infected aneurysm was negative. We continued meropenem for 6 weeks after surgery. Follow-up CT revealed resolution of the vasculitis involving the common iliac arteries without evidence of infection. As there are limited data on oral antimicrobials against *H. cinaedi*, we consulted the data for *H. pylori*. As faropenem was found to have good antimicrobial action against *H. pylori* in vitro [14], the patient was discharged on hospital day 46 with oral faropenem. He continued oral faropenem for 1 year, and his general status was stable at a 4-year follow-up.

#### Case 2

An 85-year-old woman with a past medical history of polymyalgia rheumatica treated with oral prednisolone 5 mg every other day and hypertension presented to our hospital for fever up to 38 °C and epigastric pain for 1 month that had not responded to a short course of oral cefcapene pivoxil. On admission, the patient was not in acute distress with a temperature of 36.9 °C, blood pressure of 158/48 mmHg, heart rate of 60/min, respiratory rate of 18/min, and oxygen saturation of 98% on room air. The patient was noted to have tenderness over the epigastric area but no rebound or guarding. Laboratory data showed an elevated WBC of 10,000/μL, (neutrophils 75%) and CRP of 5.27 mg/dL. Contrast-enhanced CT chest-abdomen-pelvis demonstrated increased wall thickness of the descending aorta (35 × 32 mm) and low attenuation surrounding the aorta, compatible with an infected aortic aneurysm.

**Fig. 2:**
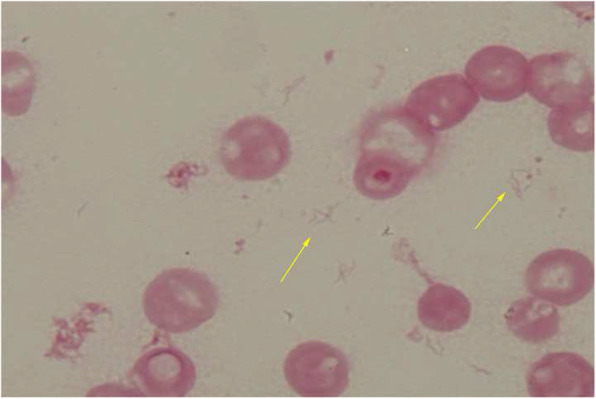
Photomicrograph of Gram stain demonstrates gram-negative spiral rods, which grew on hospital day 5 from blood cultures taken at admission

We empirically initiated ceftriaxone 2 g IV every 24 h. Her fever subsided and general status gradually improved. On hospital day 5, gram-negative spiral rods were cultured from blood samples obtained at admission (aerobic bottle, BacT/ALERT [bioMérieux]) (Fig. 2). Empirical therapy was changed to meropenem 1 g IV every 6 h. On hospital day 7, 16S rRNA gene sequencing confirmed *H. cinaedi*. The MICs for this strain tested using the same method as for Case 1 were as follows: meropenem 0.008 μg/mL, ampicillin 32 μg/mL, cefotaxime > 32 μg/mL, and levofloxacin > 32 μg/mL. Follow-up CT on hospital day 17 revealed that the infected aneurysm had diminished (33 × 31 mm). Subsequently, meropenem was switched to oral faropenem on hospital day 44. Follow-up CT on hospital day 62 revealed enlargement of the infected aneurysm (35 × 42 mm). Thereafter, urgent intravascular stenting was performed. After this procedure, the patient improved and was discharged on day 76 without further complications. The patient continued oral faropenem and was clinically stable without any sign of recurrence at the 6-year follow-up.

#### Case 3

**Fig. 3:**
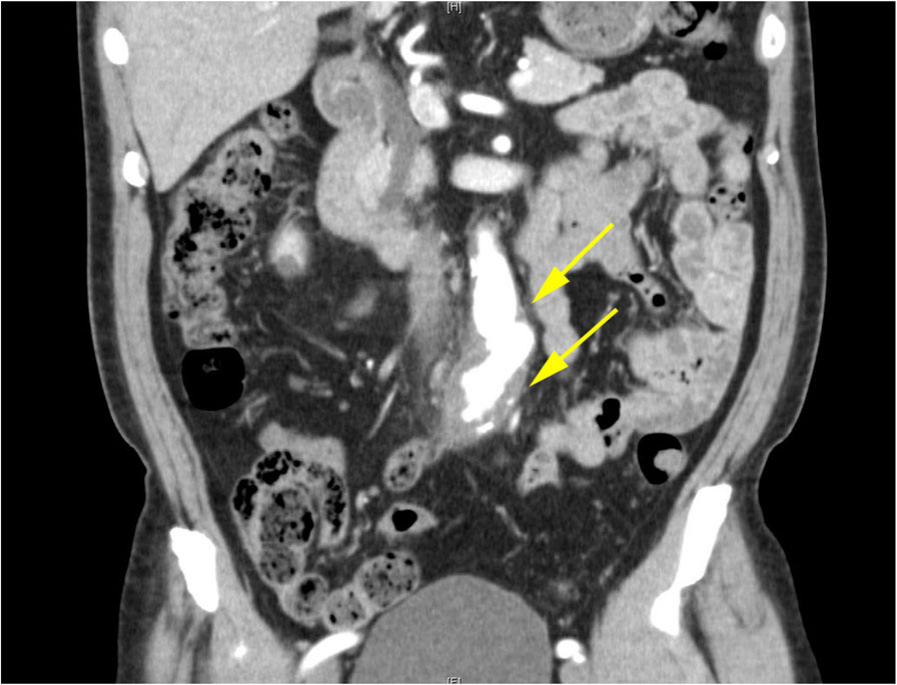
Coronal contrast-enhanced CT abdomen image demonstrates irregular wall thickening of the descending aorta having a wild, multilobulated appearance with surrounding soft tissue stranding

A 72-year-old immunocompetent man with a past medical history of hypertension and benign prostate hypertrophy presented to the emergency room with difficult and painful urination for 10 days and lower abdominal pain for 3 days followed by fever up to 38 °C 1 day prior to admission. On admission, the patient was not in acute distress with a temperature of 37.8 °C, blood pressure of 120/70 mmHg, heart rate of 89/min, respiratory rate of 18/min, and oxygen saturation of 98% on room air. The patient was noted to have tenderness of the lower abdomen without any rebound or guarding. The prostate was elastic, soft, swollen, and tender. Laboratory data showed an elevated WBC of 9800/μL, (neutrophils 78%) and CRP of 18.9 mg/dL. Contrast-enhanced CT chest-abdomen-pelvis demonstrated increased wall thickness of the descending aorta with a wild, multilobulated appearance with focal outpouching (30 × 31 mm) and fat stranding surrounding the aorta (Fig. 3), compatible with an infected aortic aneurysm.

We empirically started cefepime 1 g IV every 8 h, vancomycin IV 1 g every 12 h, and minocycline IV 100 mg every 12 h. On hospital day 12, the patient complained of worsening of lower abdominal pain, and a follow-up CT scan revealed enlargement of the aneurysm (36 × 35 mm). He underwent urgent abdominal aorta replacement on the same day. Tissue culture of the abdominal aneurysm was negative; however, 16S rRNA gene sequencing identified *H. cinaedi* from the tissue. We could not perform the susceptibility testing because *H. cinaedi* isolates could not be obtained from blood and tissue culture. The empirical therapy was changed to meropenem 1 g IV every 6 h. He was continued on meropenem for 4 weeks after surgical intervention and was discharged on hospital day 47 with oral faropenem. The patient continued oral faropenem for 3 years and was clinically stable without any sign of recurrence at the 5-year follow-up.

### Systematic review

**Fig. 4:**
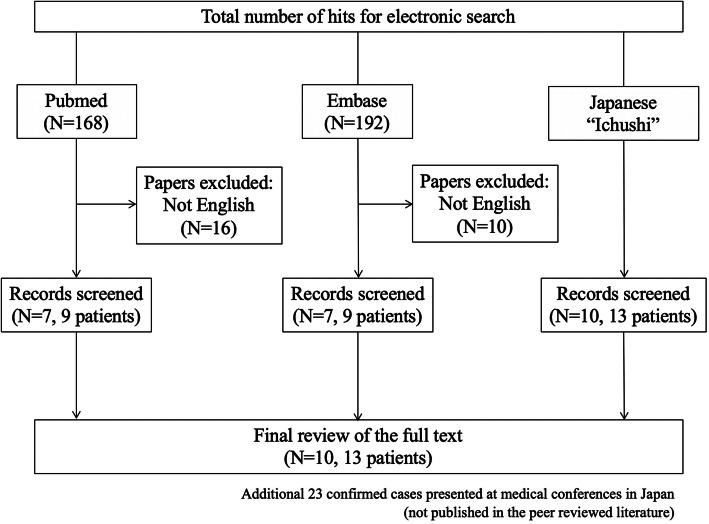
Flow chart depicts the systematic review process of this study

Two authors independently reviewed the titles and abstracts of database records, retrieved full texts for an eligibility assessment, and extracted data from these cases. A literature search was conducted in both the PubMed database (up to November 2019) using the keywords ((cinaedi) AND ((“Vascular Diseases”[Mesh]) OR (aneurysm*[TW]))) OR ((“Aneurysm, Infected”[Mesh]) AND (“Helicobacter”[Mesh] OR “Helicobacter Infections”[Mesh])) and the Embase database using the keywords (cinaedi OR ‘helicobacter cinaedi’/exp) AND (‘infected aneurysm’/exp. OR ‘aortic aneurysm’/exp. OR ‘aneurysm’) (Fig. 4). Knowing that there were several reports in Japanese papers, we have included those published only in Japanese in order to further understand the clinical characteristics of the disease by presenting more confirmed cases. In order to search for articles in Japanese, we used Ichushi, a major Japanese database, using the keywords ((cinaedi/AL)) or (“Helicobacter cinaedi”/TH)) and (((aneurysm [Japanese]/TH or aneurysm/AL)) or ((Vascular diseases [Japanese]/TH or Vascular diseases/AL)) or ((Artery [Japanese]/TH or Artery [Japanese]/AL))).

We found ten papers describing thirteen cases of infected aneurysms due to *H. cinaedi,* all from Japan [6, 15–23]. Additionally, there were twenty-three confirmed cases presented in Japan at medical conferences, though not published in the peer reviewed literature. However, for these cases detailed patient information was not available. The clinical characteristics of the thirteen published cases, including our three cases, are shown in Table 1. Among them, the most common site of infection was the abdominal aorta (at least nine patients). Only two patient received oral corticosteroids; the others were immunocompetent. Regarding management, except for two cases with conservative management, the remaining eleven patients underwent in situ grafting, extra-anatomical bypass, or intravascular stent. Except one patient with fungal infection, all patients were successfully treated without further complications.

**Table 1:** Clinical characteristics of cases of aortic aneurysm infected with *Helicobacter cinaedi*

No	Case reference	Age (years)	Sex	Published year	Underlying diseases	Chief complaint	Site of infection	Management	Antimicrobial used	Outcome
1	R. Kakuta	64	Male	2014	Hypertension	Fever, back pain	Infrarenal abdominal, L common iliac, R internal iliac, and L femoral artery	In situ grafting	Sulbactam/ampicillin 3 g/day and minocycline 100 mg/day for 25 days followed by oral amoxicillin 1500 mg/day and minocycline 200 mg/day	Complete symptom resolution
2	R. Kakuta	59	Male	2014	None	Fever, abdominal pain	Infrarenal abdominal aorta	In situ grafting	Piperacillin/tazobactam 4.5 g/day for 28 days followed by oral amoxicillin 1500 mg/day and minocycline 200 mg/day until follow-up visit	Complete symptom resolution
3	R. Kakuta	62	Male	2014	History of myocardial infarction	Low back pain	Infrarenal abdominal aorta	In situ grafting	Doripenem, 1.5 g/day for 28 days followed by oral amoxicillin 1500 mg/day and minocycline 200 mg/d, until follow-up visit	Complete symptom resolution
4	K. Niimi	60	Female	2014	Rheumatic arthritis on prednisolone, end-stage kidney disease on hemodialysis, MDR-TB	Fever, left inguinal pain	L femoral artery	Resection of the aneurysm and debridement	Ceftazidime 1 g/day for 5 days followed by imipenem-cilastatin 500 mg/day for 10 days, ampicillin/sulbactam 3 g/day for 2 weeks, and oral ampicillin/sulbactam for 1 month	Complete symptom resolution
5	T. Seto	39	Male	2014	None	Fever, chest pain	Right coronary artery (pericoronary pseudotumor)	Conservative	Ceftriaxone for 2 weeks	Complete symptom resolution
6	S. Unosawa	79	Male	2015	Hypertension	Back pain, left lower quadrant pain	Infrarenal abdominal aorta	In situ grafting with an omental wrapping	Ceftriaxone 2 g/day and gentamicin 120 mg/day, followed by sultamicillin 1250 mg/day	Complete symptom resolution
7	K. Nishida	64	Male	2015	None	Low back pain	Infrarenal abdominal aorta, L common iliac artery	In situ grafting	Meropenem and vancomycin for 8 weeks	Complete symptom resolution
8	M. Akiyama	49	Female	2016	History of myocardial infarction	Fever, back pain	Abdominal aorta, bilateral common iliac artery	In situ grafting with an omental wrapping	Piperacillin/tazobactam followed by oral amoxicillin, 1500 mg/day, and minocycline 200 mg/day for 3 months	Complete symptom resolution
9	J. Inagaki	80	Male	2017	None	Fatigue	Infrarenal abdominal aorta, bilateral common iliac artery	Extra-anatomical bypass	Meropenem and levofloxacin (dose not available) for 6 weeks followed by minocycline and rifampicin for more than 18 months	Complete symptom resolution with continued antimicrobials
10	K. Kushimoto	68	Male	2017	Hypertension, hyperuricemia	Fever, chest and back pain	Distal aortic arch, thoracic aorta	In situ grafting with an omental wrapping	Levofloxacin 250 mg/day for 19 days followed by minocycline 200 mg/day	Complete symptom resolution with continued antimicrobials
11	Y. Kanno	73	Male	2018	History of abdominal aortic aneurysm, colon polyp	Back pain	Abdominal aorta	Conservative first, followed by in situ grafting 3 months later	Meropenem 3 g/day and vancomycin 1 g/day for 7 days followed by levofloxacin 500 mg/day for 5 days, and oral sultamicillin 1200 mg/day for 3 months (relapsed) ampicillin/sulbactam 6 g/day for 12 days followed by oral sultamicillin 1200 mg/day (duration not available)	Complete symptom resolution
12	Y. Kanno	72	Male	2018	None	Fever	Thoracic aorta	In situ grafting	Meropenem 3 g/day and vancomycin 1 g/day followed by ampicillin/sulbactam 6 g/day	Deceased (fungal infection)
13	S. Nakao	65	Male	2018	None	Fever and right neck pain	R common carotid artery	Conservative	(1st) Meropenem 6 g/day followed by ceftriaxone 4 g/day for 2 weeks and oral minocycline 200 mg/day for 2 weeks (2nd) Ceftriaxone 4 g/day for 2 weeks followed by oral ampicillin 1.5 g/day and doxycycline 400 mg/day for 6 weeks	Complete symptom resolution
14	T. Matsuo	77	Male	2020	Hypertension	Fever and left chest pain	Aortic arch and bilateral common iliac artery	In situ grafting	Meropenem followed by faropenem 1200 mg/day for 1 year	Complete symptom resolution
15	T. Matsuo	85	Female	2020	Polymyalgia rheumatica and hypertension	Epigastric pain	Descending aorta	Intravascular stent	Meropenem followed by faropenem 1200 mg/day	Complete symptom resolution with continued antimicrobials
16	T. Matsuo	72	Male	2020	Hypertension and benign prostate hypertrophy	Fever and lower abdominal pain	Bilateral common iliac artery	In situ grafting	Meropenem followed by faropenem 1200 mg/day for 3 years	Complete symptom resolution

*L* Left, *R* Right, *MDR-TB* Multidrug-resistant tuberculosis

## Discussion and conclusions

This is the first systematic review of infected aortic aneurysms caused by *H. cinaedi*. The mechanisms underlying infected aortic aneurysms remain unknown. However, four main mechanisms have been hypothesized that result in infection of the arterial wall: 1) the development of infected aneurysms secondary to septic microemboli of the vasa vasorum, 2) extension from a contiguous infected focus, 3) hematogenous seeding of the intima during bacteremia originating from a distant infection, and 4) trauma to the arterial wall with contamination [24]. Recently, Araoka et al. reported that bacterial translocation from the intestinal tract could be a route that leads to *H. cinaedi* bacteremia [25]. The promotion of atherosclerosis by *H. cinaedi* has also been reported [26, 27]. In our three cases, none had evidence of infection with *H. cinaedi* at distal sites, extension from a contiguous infected focus, or a history of trauma; therefore, the development of infected aneurysms secondary to septic microemboli of the vasa vasorum or bacterial translocation from the intestinal tract were considered the most likely mechanisms.

There is a lack of data on susceptibility testing, and standard breakpoints of antimicrobial agents for *H. cinaedi* have not been defined. Previous studies reported that susceptibility testing for *H. cinaedi* isolates has been conducted using the agar dilution method [28, 29]. Low MIC values were generally reported for *H. cinaedi* strains for carbapenems, aminoglycosides, and tetracycline (MIC_90_ ≤ 1 μg/mL) [5, 30], moderate MIC values for ampicillin (MIC_90_ = 16 μg/mL), cefepime (MIC_90_ = 8 μg/mL), and ceftriaxone (MIC_90_ = 8 μg/mL) [5], and high MIC values for erythromycin (MIC_90_ > 64 μg/mL) [31]. Prior to 2000, low MICs of ciprofloxacin and macrolides were reported for *H. cinaedi*; however, the majority of *H. cinaedi* isolates have gained resistance since the early 2000s because of the increased use of these antimicrobials [30]. As the agar dilution method is not available in all institutes, the Etest could be an alternative method; however, it can be inaccurate because of the unclear edges around the growth inhibition zone [5]. Although there are no guidelines for recommended antimicrobial treatment, there has been varied used of beta-lactams such as penicillin, cephalosporin, and carbapenem [2, 32]. Monotherapy versus combination therapy has not been fully investigated. Further studies exploring the antimicrobial susceptibility profiles are warranted.

Except for one case, all of the reviewed cases underwent open repair (OR). OR is considered the gold standard with weak evidence supporting its superiority compared with endovascular aortic repair (EVAR). The optimal treatment of infected aneurysms remains unknown, although an increasing number of trials have reported EVAR as an alternative treatment for infected aortic aneurysms, with improved short-term survival compared with OR without the associated higher incidence of serious infection-related complications or reoperations [33]. In contrast, Luo et al. reported that persistent infection after EVAR does occur and is often fatal without surgical treatment [34]. The choice between OR or EVAR should be weighed against the risks and benefits for each patient.

The optimal duration of antimicrobial therapy for *H. cinaedi* infection also remains unknown. In addition to IV antimicrobials for at least 6 weeks after surgery, some experts recommend that patients continue oral antimicrobials after discharge for at least 6 months whereas others recommend lifelong therapy [8]. All of our patients continued antimicrobials for at least for 1 year.

Regarding the incidence and prevalence of *H. cinaedi*, all published reports of infected aneurysms caused by *H. cinaedi* are from Japan. The number of reports of *H. cinaedi* overall infection, including bacteremia and skin and soft tissue infection, has been increasing over the last 10 years, especially in Japan. It is thought that there are three main reasons for this. First, as *H. cinaedi* is becoming increasingly recognized in Japan, many institutions are extending the incubation period for blood cultures when clinicians suspect *H. cinaedi* infection. *H. cinaedi* usually grows slowly, and one clinical research study from Japan revealed that approximately 50% of *H. cinaedi* bacteremia would probably have been overlooked had the duration of monitored blood cultures been limited to 5 days [35]. After this report, many institutes extended the incubation period. Another hypothesis is that *H. cinaedi* may be related to Japanese-specific cultural behaviors such as eating raw fish as sushi and sashimi followed by colonization of *H. cinaedi* in the intestine. However, this is perhaps less likely given the increasing worldwide popularity of Japanese cuisine. Finally, some institutions have changed blood culture products, moving from BacT/ALERT to BACTEC (BD Diagnostics, Sparks, MD), and this could also be contributing to the increased detection of *H. cinaedi* [36]. New BacT/ALERT plus bottles that modify the antimicrobial-absorbing materials in the blood culture media could also have contributed to the improved detection [37, 38]. However, the true reason why *H. cinaedi* infections are increasing in Japan is as yet unknown. As these detections were made using the blood culture system described above for extended cultures and 16S rRNA gene sequencing in tissues, the sensitivity and specificity of the techniques for diagnosis and detection should be analyzed in the future. Furthermore, studies on the true mechanisms of *H. cinaedi* infections and its incidence and prevalence are warranted.

In summary, aortic aneurysms infected with *H. cinaedi* are rare and occur in immunocompetent as well as immunocompromised individuals. Clinicians should suspect *H. cinaedi* as a possible causative pathogen in patients with infected aortic aneurysms and extend the incubation period for blood cultures. The optimal choice of antimicrobials for *H. cinaedi,* the duration of therapy for infected aneurysms, and the optimal intervention regarding OR versus EVAR should be investigated in further studies.

## Data Availability

Not applicable.

## Abbreviations

*H. cinaedi*: *Helicobacter cinaedi*
WBC: White blood cell
CRP: C-reactive protein
CT: Contrast whole trunk computed tomography
IV: Intravenously
MIC: Minimum inhibitory concentration
OR: Open repair
EVAR: Endovascular aortic repair
*C. trachomatis*: *Chlamydia trachomatis*
